# ANKYLOGLOSSIA AND ITS INFLUENCE ON GROWTH AND DEVELOPMENT OF THE STOMATOGNATHIC SYSTEM

**DOI:** 10.1590/1984-0462/;2017;35;2;00016

**Published:** 2017

**Authors:** Livia Eisler Pompéia, Roberta Simoni Ilinsky, Cristina Lúcia Feijó Ortolani, Kurt Faltin

**Affiliations:** a Universidade Paulista (UNIP), São Paulo, SP, Brasil.

**Keywords:** lingual frenum, growth and development, child

## Abstract

**Objective:**

To critically examine the existing Brazilian and International scientific literature regarding the influence of short lingual frenulum over growth and development of the stomatognathic system, as well as how it impacts the achievement of the shape-function balance.

**Data sources:**

An electronic literature search was conducted in databases, including MEDLINE/PubMed, Google Scholar, LILACS, SciELO, and ScienceDirect, using the key words “lingual frenum” and “development”, as well as their equivalents in Brazilian Portuguese. The literature search yielded 51 papers published between January 1997 and the present date; 14 articles of clinical trials were selected for meeting the inclusion criteria and were read in full.

**Data synthesis:**

The integrated literature review supported the proposition that some malocclusions are closely related to the presence of ankyloglossia and, although very few clinical trials on this topic have been published so far, there is a consensus among authors concerning the negative effects of functional imbalances over the stomatognathic system’s proper growth and development. Half of the studies found state that surgical interventions for releasing the lingual frenum are both safe and effective, concerning improvement in breastfeeding scores. Moreover, 4 out of the 14 studies included in this integrated review, report a negative influence of ankyloglossia over the orofacial muscular system.

**Conclusions:**

There is a consensus among the authors concerning the negative effects of lingual frenulum’s anatomic and functional alterations over craniofacial growth and development. The opinion about the early surgical intervention, however, is not unanimous.

## RESUMO

**Objetivo:**

Avaliar criticamente os artigos existentes na literatura brasileira e estrangeira acerca da influência do frênulo lingual encurtado sobre o crescimento e desenvolvimento do sistema estomatognático, bem como sobre a conquista do equilíbrio forma-função.

**Fontes de dados:**

A busca eletrônica nas bases MEDLINE/PubMed, Google Scholar, LILACS, SciELO e ScienceDirect com os descritores “freio lingual” e “desenvolvimento” e seus equivalentes em inglês resultou inicialmente em 51 resultados de trabalhos publicados entre janeiro de 1997 e a presente data. Após aplicados os critérios de exclusão, 14 artigos de estudos clínicos foram selecionados para a leitura completa.

**Síntese dos dados:**

A revisão integrativa da literatura ajudou a confirmar a proposição de que algumas maloclusões estão intimamente relacionadas à anquiloglossia. Ainda que a quantidade de estudos clínicos publicados até a atualidade seja pequena, há um consenso entre os autores acerca do efeito negativo de desequilíbrios funcionais sobre o crescimento e o desenvolvimento corretos do sistema estomatognático, tanto que a metade dos estudos pesquisados relata que intervenções cirúrgicas para a liberação do frênulo lingual são seguras e eficazes no que diz respeito à melhora na amamentação. Ainda, dos 14 estudos incluídos nesta revisão, 4 reportam influências negativas da anquiloglossia sobre a musculatura orofacial.

**Conclusões:**

Há um consenso entre os autores acerca dos efeitos negativos das alterações anatômico-funcionais do frênulo lingual sobre o crescimento e o desenvolvimento craniofacial, ainda que a opinião sobre a intervenção cirúrgica precoce não seja unânime.

## INTRODUCTION

The lingual frenulum is a fibrodense conjunctive fold, occasionally made up of superior fibers of the genioglossus muscle, which are inserted in the ventral tongue, between the apex and the middle third, and in the floor of the mouth, which may be between the lingual carunculi or previously displaced to the lower alveolar ridge.^1^ Ankyloglossia, or shortening of the free lingual portion, is an anatomical condition characterized by the restriction of tongue movement, which may have a strong impact on its function, also interfering in the shape of the dental arches and their consequent occlusion. Such condition occurs in 4-16% of neonates, with a preference for male patients in a proportion of 2.5:1.^2^

The tongue originates from the first, second, and third pharyngeal arches during the fourth week of gestation. In this phase, grooves are formed laterally to the structure, so that it can move freely, except for the region adhered by the lingual frenulum, initially at the apex of the tongue. As development occurs, the cells of the frenulum undergo apoptosis and tend to migrate distally to the medial region of the lingual dorsum. At this time, there may be interferences in cell control and the migration may be incomplete, or even not occur, establishing the condition of ankyloglossia.^1^

Several authors have described relations between malocclusions and functional disorders of the oral cavity and its adjacent muscles, as well as the importance of establishing a functional balance of the stomatognathic system to achieve the stability of the form. Natural breastfeeding plays an important role in the maturation of the perioral musculature and, therefore, in the development of correct breathing, swallowing and, subsequently, occlusion.^3^

Praetzel et al.^4^ retrospectively studied 595 patients aged between 1 and 14 years and related the findings of myofunctional disorders and the time of natural breastfeeding. The authors reported that 54% of the sample showed some changes in the stomatognathic system (mouth breathing, open bite, atypical swallowing, ankyloglossia), of which almost 70% were breastfed naturally for less than 6 months. With these findings, they could conclude that the growth and development of the face depend on the correct function performance of the entire stomatognathic system and, by analogy, dysfunctions in respiration, suction, swallowing, chewing, and phonation are closely related to changes in the shape of the arches and their relationship with their respective bone bases. They also added that prolonged breastfeeding should be encouraged because it is clearly important in the prevention of facial dysfunction, as it generates the neural stimuli that will modulate the baby’s craniofacial growth and development.

One of the central problems of a strongly inserted lingual frenulum is the need for adaptation to breastfeeding. During breastfeeding, some masticatory muscles begin to mature and position, such as the temporal (activated in the retrusion of the mandible), the lateral pterygoid (requested in the propulsion), the milo-hyoid (main responsible for swallowing), and the masseters (activated in the suction mechanics), while the orbicularis of the upper and lower lips guide the growth and development of the anterior region of the stomatognathic system,^5^ ^,^ ^6^ which should work in full neuromotor balance for the mechanics of chewing and swallowing to be efficient. According to Van der Laan,^5^ the muscular effort that occurs in breastfeeding is a physical preparation for the future masticatory function. The various repetitions of protrusive and retrusive movements throughout the day are capable of positively stimulating the temporomandibular joints for the anteroposterior growth of the mandible, as described by Stutzmann and Petrovic^7^ in 1990, thus preventing many distoclusions.

Normal mature swallowing occurs when the tip of the tongue is compressed against the incisive papilla, with its dorsum pushing the alimentary bolus through the hard palate toward the oropharynx, with posterior teeth in maximal intercuspidation and orbicularis muscles of the upper and lower lip and mentonian relaxed and promoting lip sealing. A review of the literature indicates different types of atypical swallowing, all of which are potentially responsible for the installation of malocclusions or for treatment relapse, because they characterize situations in which an imbalance between the buccinator, pterygoids, masseter, and temporal muscles.^2^

Most studies on the anatomical-functional alterations of the lingual frenulum are concerned with establishing a relationship between the pathologies and the quality of natural breastfeeding or phonetic disorders, a problem with which frenulum shortening is already related. The objective of this integrative review was to find evidence that links this condition to the establishment of changes in craniofacial development.

## METHOD

This is an integrated review that selected and analyzed bibliographic materials available in the following electronic databases: Literatura Latino-Americana e do Caribe (LILACS), International Literature in Health and Biomedical Sciences (PubMed/MEDLINE), Google Scholar, Scientific Electronic Library Online (SciELO), and Science Direct, using the terms “lingual frenulum” and “development” and their equivalents in the Portuguese language as descriptors, in order to elaborate an objective critical analysis on the subject.

The review comprised the period from 1997 to 2016, during which there were 51 publications related to the subject. First, all titles and abstracts were analyzed, and, applying the exclusion criteria stipulated by the authors, 37 articles were eliminated from the analysis because they were clinical cases, literature reviews or publications with incomplete abstracts, or without clarity of objectives and articles whose language of publication was not Portuguese or English. After applying the criteria, 14 articles were read and analyzed in full (Chart 1).

**Table 1:**
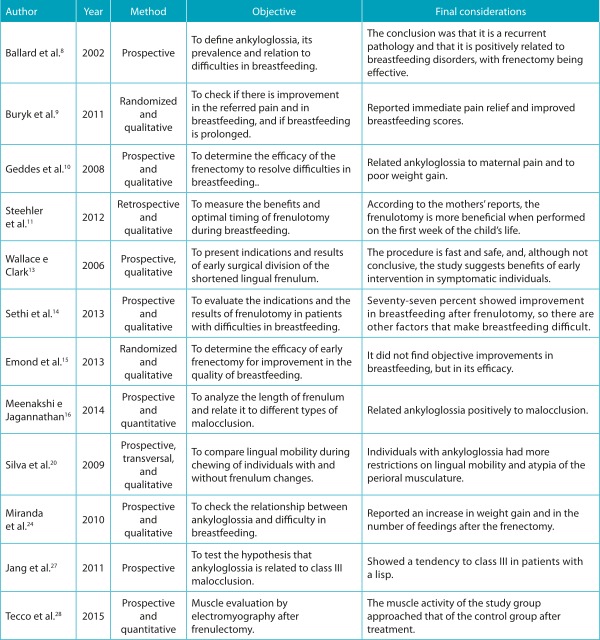
Summary of the information extracted from the articles analyzed in this review.

## LITERATURE REVIEW AND DISCUSSION

Recently, a law was passed in Brazil that requires the application of the evaluation protocol on the lingual frenulum of babies born in all hospitals and maternities, public or private. This procedure aims at the prevention of complications that make breastfeeding difficult due to deficiencies in the sucking and swallowing functions, which is associated with low weight gain, as well as early weaning, and may be related to ankyloglossia, as referred to in the literature.^8^ ^,^ ^9^ ^,^ ^10^ ^,^ ^11^ ^,^ ^12^ ^,^ ^13^

In a clinical study conducted by Ballard et al.^8^ in 2002, it was found that 4% of infants with breastfeeding difficulties had some degree of shortening of the lingual frenulum, and frenectomy proved to be effective in improving the baby’s suckling on the breast and as pain relief reported by mothers in 100% of cases, which is consistent with what Buryk et al.^9^ reported in their randomized clinical study on early frenectomy, and its immediate positive outcomes in breastfeeding scores. Geddes et al.^10^ were able to clinically prove, through ultrasound imaging in the submental region, the increased flow of milk transferred from the mother’s breast to the oral cavity in 100% of the babies after the frenectomies.

Steehler et al.,^11^ in a retrospective study from 2012, reported that 86% of the mothers of patients who underwent frenulotomy before completing one week of life noticed large improvements in breastfeeding. Among the mothers of children submitted to the procedure after the first week of life, the improvement in breastfeeding was reported by 74%, suggesting greater efficiency in early intervention.

In the following year, Sethi et al.^14^ prospectively evaluated 85 children aged between 3 and 120 days, who presented difficulties related to breastfeeding. The patients were submitted to the frenulotomy and 52 of them, evaluated over 5 months, through a telephone interview with questions about the pain reported by the mother when breastfeeding, the number of suckles a day, the sucking noise, and the volume of milk flow. The results showed improvement in breastfeeding within two weeks after the procedure for 77% of the mothers, indicating a relationship between ankyloglossia and difficulties in breastfeeding, which, however, is not the only cause of difficulties. In that same year, Emond et al.^15^ conducted a similar study, but with the inclusion of a control group. In the latter study, although the maternal perception of breastfeeding efficacy was positive, no objective improvement in breastfeeding was observed after precocious frenectomy.

Contrary to what the previous literature suggested,^16^ ^,^ ^17^ ^,^ ^18^ ^,^ ^19^ Martinelli et al.^18^ evaluated 71 infants from the first to the twelfth month of life regarding the characteristics and location of the brake insertion, both on ventral tongue and on the floor of the mouth, and clinically proved that there is no change or migration of the insertion of superior fibers from the genioglossus during the first year of the baby’s life, since in 100% of the patients there was no change in the position, thickness, or length of the structure. Silva et al.^20^ also observed clinically that subjects with ankylosis showed about 5.5 times more restrictions of lingual mobility and muscle atypia during mastication than those with their normally inserted lingual frenulum. Both studies justify the early indication of the surgical intervention as soon as the diagnosis of ankyloglossia is made, although the findings in the literature on how to proceed clinically in these cases are quite divergent.^21^ ^,^ ^22^ ^,^ ^23^ ^,^ ^24^ ^,^ ^25^ ^,^ ^26^ There is no consensus on the real need for surgical treatment, what time or technique to adopt in case of opting for surgery, or even on which professional is qualified to perform it.

Jang et al.^27^ and Meenakshi e Jagannathan,^16^ in clinical studies, positively correlated changes in the lingual frenulum and the presence of malocclusions. The last authors classified 30 patients aged 12-16 years into three groups according to the type of malocclusion. Horizontal and vertical measurements of the length of the lingual brake and the opening width of the mouth were taken in two moments: maximum opening and maximum opening with the tip of the tongue touching the incisive papilla. The mean length of the lingual frenulum for class I patients was 25% lower than those of Class III. Although these authors report that structure migration to puberty occurs (a fact challenged by Martinelli et al.^18^), the authors suggest that early diagnosis and treatment of ankyloglossia provide a better prognosis in the treatment of class III malocclusions. Similarly, Jang et al,^27^ in 2011, clinically observed 150 patients divided equally between the three types of malocclusions and established a direct method (by specific rule) and an indirect method (maximum open mouth width with and without touch of the incisive papilla) to measure the lingual frenulum. These authors concluded that class III patients showed significant changes in the length of the frenulum and the reduction of opening amplitude in relation to the other groups, thus establishing a possible relationship between the insertion of the structure and the distoclusion.

In a recent clinical study, Tecco et al.^28^ assessed, by means of surface electromyography, 24 individuals with class I malocclusion in the mixed dentition. The sample was divided into a control group, composed of 11 individuals with normal lingual brakes, and a study group, with 13 individuals with ankyloglossia. The study consisted in evaluating the muscular activity of the masseter, temporal, orbicular, and submental muscles in three moments: at the beginning of the treatment (T0), one month (T1), and six months (T2) after the release of the lingual brake in the study group. The results of the electromyographic potentials were compared and the researchers could verify a difference between the muscle activity of the study group and the T0 control, but similarities between them in T2, concluding that the lingual frenectomy was able to influence the function of the orofacial muscles studied.

Ankyloglossia can then be associated with occlusal problems and craniofacial development, since the low posture of the tongue was related to the installation of class III malocclusion.^16^ ^,^ ^27^ Still, from a more complex point of view, the correction of the resting position of the tongue improves the position of the hyoid bone, decreasing the muscular forces on the mandible in order to rotate it back and down and, in contrast, the positioning of the tongue on the oral floor lowers the hyoid bone, distending the musculature inserted to the mandible, which favors its rotation clockwise, resulting, for example, in anterior open bite.

The low number of publications relating ankyloglossia and development over the 19 years studied is highlighted, although most of the findings date from the last decade, indicating an increase in interest in the topic. That is, it is still a field with much room for research, both clinical and academic, in order to provide a better theoretical basis to professionals in the area.

It is possible to conclude that, although there are divergences on the indication of the clinical-surgical intervention for the correction of ankyloglossia, there is a consensus in the literature about the negative effect of functional imbalances caused by ankyloglossia on the correct growth and development of the stomatognathic system. All the researched authors report the need to establish neuromuscular balance in order to achieve stable esthetic-functional goals.
